# Leveraging Digital Technology in Conducting Longitudinal Research on Mental Health in Pregnancy: Longitudinal Panel Survey Study

**DOI:** 10.2196/16280

**Published:** 2021-04-27

**Authors:** Beth McGee, Marie Leonte, Kevin Wildenhaus, Marsha Wilcox, Jenna Reps, Lauren LaCross

**Affiliations:** 1 BabyCenter, LLC San Francisco, CA United States; 2 Janssen Research & Development, LLC Titusville, NJ United States

**Keywords:** digital, longitudinal, pregnancy, postpartum, perinatal, panel, study design, mental health

## Abstract

**Background:**

Collecting longitudinal data during and shortly after pregnancy is difficult, as pregnant women often avoid studies with repeated surveys. In contrast, pregnant women interact with certain websites at multiple stages throughout pregnancy and the postpartum period. This digital connection presents the opportunity to use a website as a way to recruit and enroll pregnant women into a panel study and collect valuable longitudinal data for research. These data can then be used to learn new scientific insights and improve health care.

**Objective:**

The objective of this paper is to describe the approaches applied and lessons learned from designing and conducting an online panel for health care research, specifically perinatal mood disorders. Our panel design and approach aimed to recruit a large sample (N=1200) of pregnant women representative of the US population and to minimize attrition over time.

**Methods:**

We designed an online panel to enroll participants from the pregnancy and parenting website BabyCenter. We enrolled women into the panel from weeks 4 to 10 of pregnancy (Panel 1) or from weeks 28 to 33 of pregnancy (Panel 2) and administered repeated psychometric assessments from enrollment through 3 months postpartum. We employed a combination of adaptive digital strategies to recruit, communicate with, and build trust with participants to minimize attrition over time. We were transparent at baseline about expectations, used monetary and information-based incentives, and sent personalized reminders to reduce attrition. The approach was participant-centric and leveraged many aspects of flexibility that digital methods afford.

**Results:**

We recruited 1179 pregnant women—our target was 1200—during a 26-day period between August 25 and September 19, 2016. Our strategy to recruit participants using adaptive sampling tactics resulted in a large panel that was similar to the US population of pregnant women. Attrition was on par with existing longitudinal observational studies in pregnant populations, and 79.2% (934/1179) of our panel completed another survey after enrollment. There were 736 out of 1179 (62.4%) women who completed at least one assessment in both the prenatal and postnatal periods, and 709 out of 1179 (60.1%) women who completed the final assessment. To validate the data, we compared participation rates and factors of perinatal mood disorders ascertained from this study with prior research, suggesting reliability of our approach.

**Conclusions:**

A suitably designed online panel created in partnership with a digital media source that reaches the target audience is a means to leverage a conveniently sized and viable sample for scientific research. Our key lessons learned are as follows: sampling tactics may need to be adjusted to enroll a representative sample, attrition can be reduced by adapting to participants’ needs, and study engagement can be boosted by personalizing interactions with the flexibility afforded by digital technologies.

## Introduction

Mental health and mood disorders, such as depression and anxiety, can cause negative outcomes for women [1] and can lead to health and developmental problems for their offspring [2]. A better understanding of perinatal mental health is needed to help families lead healthier lives. To observe the totality of perinatal depression, it is important to include women early in pregnancy and obtain repeated assessments starting at this early stage and into the postnatal period. The challenges to accomplish this include lack of access to pregnant women before they have been assessed in clinical settings, where many pregnancy studies recruit participants, and difficulty maintaining cooperation throughout pregnancy and into the postpartum period.

An additional roadblock when researching perinatal depression is the reluctance of pregnant women to participate in scientific or medical studies, as pregnant women exhibit lower cooperation rates than the general population of women [3]. Concern for the fetus and pregnancy and lack of connection with the research goals contribute to this reduced cooperation [4]. In addition, enrolling a representative pregnant population may be difficult, as research has shown that African American pregnant women are less willing to take surveys associated with medical research; this can challenge researchers to construct and maintain representative samples [3]. It has been shown that building trust is pivotal when conducting research among pregnant women and necessary to increase participation [5].

There have been successful longitudinal cohort studies conducted in Europe and Asia. The Maternal Anxiety in Relation to Infant Development (MARI) Study recruited 483 pregnant women at weeks 10 to 12 from community clinics in Dresden, Germany [6]. The Growing Up in Singapore Towards healthy Outcomes (GUSTO) Study recruited 1247 women during their first clinical visit of pregnancy (ie, <14 weeks) and followed them through birth and to 36 months postpartum [7]. Our study aimed to conduct longitudinal research with a panel that was representative of US women giving birth, starting from week 4 of pregnancy.

BabyCenter was a suitable platform to recruit a large population of pregnant women into a panel that was similar to the profile of pregnant women in the United States. It is a digital resource for pregnancy and parenting information that reaches 3 in 4 pregnant women in the United States [8]. Pregnant women begin accessing the BabyCenter website early in pregnancy, often before their first prenatal visit; over three-quarters of BabyCenter pregnancy website registrations occur during the first trimester, with weeks 4, 5, and 6 of pregnancy seeing the largest percentage of registrations, according to BabyCenter’s internal tracking data.

We designed and conducted a comprehensive longitudinal study of perinatal mental health among a large panel of women reflective of all US women giving birth. We administered frequent assessments using electronic patient-reported outcome assessments beginning early in pregnancy and through the postnatal period. The goal was to minimize participant attrition and generate a well-characterized data set to further the knowledge of perinatal mood disorders. The aim of this paper is to demonstrate methods used to recruit pregnant participants into an online panel to ensure we obtained a large representative sample and describe how we reduced attrition. We also describe lessons learned that could improve future online panel recruitment and retention for difficult-to-survey populations.

## Methods

### Recruitment and Enrollment

We conducted a longitudinal study with a population-based sample of pregnant women, aged 18 years and older, in the United States, from early in pregnancy to 12 weeks postpartum. The sampling frame for this work was the BabyCenter website. Additional inclusion criteria for the study were as follows: weeks 4 to 10 of pregnancy (Panel 1) or weeks 28 to 33 of pregnancy (Panel 2) and not currently participating in other research studies.

**Figure 1:**
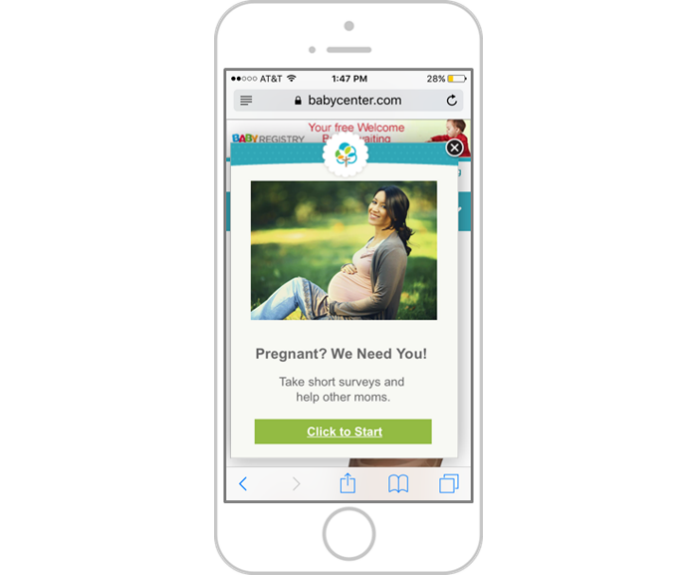
Survey floater invitation on a mobile device. The advert shows a smiling pregnant lady with the text “Pregnant? We Need You! Take short surveys and help other moms.”

From August 25 to September 19, 2016, BabyCenter website visitors were selected at random and shown a floating invitation during their website experience (see Figure 1). Invitations used friendly language, a description of incentives for participation, and an altruistic approach, as this has been shown to be a key motivator for pregnant women to participate in research [3]. The recruitment goal was to enroll 1200 participants in a 6-week period. The goal of 1200 participants was determined with consideration to power calculations, anticipated time frames for recruitment, and an effort to sample a similar or larger panel size than had been demonstrated in previous longitudinal studies of pregnancy and mental health.

Participants enrolled in the study on their own, without support of study researchers, within the digital survey environment upon completion of a screening and enrollment baseline assessment. They were provided detailed information about the study’s timing, protocol, and incentives. Participants’ consent was obtained via digital agreement within this same baseline assessment. We had New England Institutional Review Board approval to complete this work.

Recruitment strategies were designed to balance the sample to closely match the demographic profile of US women giving birth as reported by government agencies [9]. To this end, adjusting specific digital sampling parameters either increased or decreased the proportion of participants in certain demographic groups.

### Study Content

The baseline assessment included screening questions, health history, demographic profiling, pregnancy health assessment, and information about recent life events. The final assessment, administered at 12 weeks postpartum, measured the birth experience. The study contained a battery of standardized psychometric assessments relevant to the topic of perinatal mood disorder that repeated at set intervals throughout the course of the study, measuring anxiety, stress, and obsessive-compulsive tendencies (see Table 1). The study employed the Edinburgh Postnatal Depression Scale (EPDS), the accepted standard measure of mood in the perinatal period, as the primary indicator of major depressive disorder [10]. We excluded the suicidality item in the EPDS scale due to the study’s lack of provision for intervention for women who may have self-identified to be at risk.

**Figure 2:**
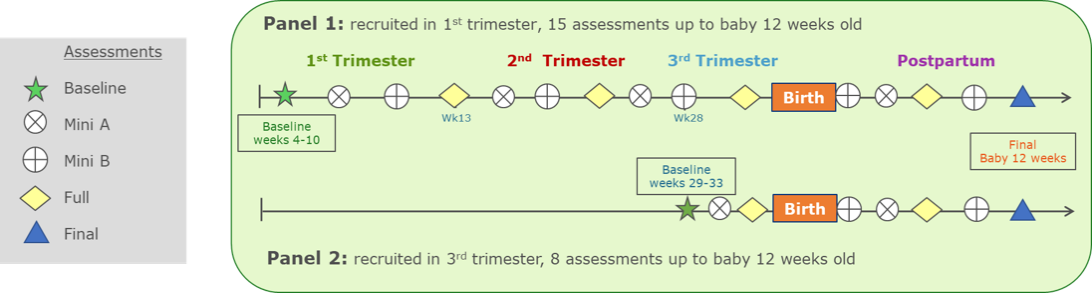
Assessment protocol overview.

There were two iterations of short-form assessments, labeled *Mini A* and *Mini B*, and one iteration of a long-form assessment, labeled *Full*. Each of the three total assessment types contained varied sets of psychometric scales alternating in the study protocol to maximize the types of information collected, provide measurements at regular intervals of 1 to 4 weeks, and reduce monotony and response burden (see Figure 2 and Multimedia Appendix 1).

Panel 1 had the opportunity to complete a total of 15 assessments including the one at baseline, while Panel 2 could complete a total of 8 assessments including the one at baseline.

**Table 1:** Collected data, assessment instruments, and time points of measurements.

Collected data or assessment instrument	Baseline^a^	Mini A^b^ (short form)	Mini B^c^ (short form)	Full^d^ (long form)	Final^e^
Health history	✓^f^				
Demographic profile	✓				
9-item Edinburgh Postnatal Depression Scale	✓		✓	✓	✓
4-item Perceived Stress Scale	✓		✓	✓	✓
6-item State-Trait Anxiety Inventory	✓		✓	✓	✓
4-item PROMIS^g^ Emotional Support	✓		✓	✓	✓
7-item Generalized Anxiety Disorder	✓			✓	✓
18-item Obsessive-Compulsive Inventory-Revised				✓	✓
4-item PROMIS Pain Interference		✓			
4-item PROMIS Sleep Disturbance		✓			
8-item PROMIS Sleep-Related Impairment		✓			
4-item PROMIS Anxiety		✓			
2-item Patient Health Questionnaire		✓			
14-item Perinatal Post-Traumatic Stress Disorder Questionnaire-Modified					✓
Birthing data					✓

^a^Data were collected at pregnancy weeks 4-10 and 29-33.

^b^The Mini A (short-form) instrument contained five psychometric questions and, on average, took 5 minutes to complete. Data were collected at pregnancy weeks 6, 7, 9-11, 15, 25, 32, and 34 and postpartum week 1.

^c^The Mini B (short-form) instrument contained four psychometric questions and, on average, took 5 minutes to complete. Data were collected at pregnancy weeks 9, 11, 12, 18, and 28 and postpartum +2 days and week 8.

^d^The full (long-form) survey contained six psychometric questions and, on average, took 7 minutes to complete. Data were collected at pregnancy weeks 12, 13, 21, 32, and 35 and postpartum week 4.

^e^Data were collected at postpartum week 12.

^f^Check marks indicate that the indicated data were collected or the indicated version of the assessment instrument was conducted at this time point.

^g^PROMIS: Patient-Reported Outcomes Measurement Information System.

Assessments were meant to create a panel experience that was enjoyable and stress free. At the beginning of every assessment, respondents were asked two or three pregnancy or parenting lifestyle questions unrelated to the psychometric assessments. These included questions about pregnancy, diet, the baby’s sex, and preparation for the baby’s arrival. The inclusion of these lifestyle questions was intended to foster participant engagement and counterbalance the serious nature of the psychometric assessments (see Multimedia Appendix 2).

Assessments were optimized for mobile devices for easy viewing and completion of questions. All assessments were administered through the Qualtrics platform, and respondent data were stored in the secure environment of Qualtrics Target Audience, which is currently known as Qualtrics Core XM [11].

### Assessment Invitations

Participants received invitations to complete assessment surveys by email. The assessment interval was an established protocol, but the actual date a participant was invited to complete a survey was customized for each participant based on the date of enrollment and the pregnancy week at baseline. We created an application programming interface (API) within Qualtrics that enabled unique protocol dates for each participant. The API distributed automated email invitations, reminders, and incentives. The API deployed reminders as needed, with up to three reminders delivered over the duration of each survey window, which was typically 7 days. This volume and timing of communication was intended to maximize response but not overburden participants with emails.

A challenge when studying a pregnant population into the postnatal period is that the birth date of the baby is an unknown time variable that cannot be pre-established. To address this, as pregnancy progressed into the late third trimester, we invited women to complete a birth survey to confirm the arrival of the baby. Participants received birth survey invitation emails through week 42 of pregnancy. Completing the birth survey initiated a new protocol within the API, with the baby’s birth date now serving as the baseline date for initiating the postnatal surveys.

### Incentives

Declining participation in epidemiologic studies has necessitated the use of monetary incentives; this is an accepted method to increase cooperation [12]. This study’s duration—9 to 11 months for most participants—required an incentive strategy to head off attrition. Participants in Panel 1 had the opportunity to earn a total of US $180 in e-gift cards over the course of the study, and participants in Panel 2 had the opportunity to earn a total of US $125 in e-gift cards over the course of the study. When an incentive was attained, it was fulfilled automatically by the API via email, making it easy for participants to track and redeem their rewards.

We included a second incentive to help maintain participation through the study’s end: a sweepstakes to encourage participants to complete the maximum assessments. Separate US $1000 sweepstakes were offered for Panel 1 and Panel 2 participants. A respondent in Panel 1 who completed all 15 assessments would increase their odds of winning by earning 15 entries. A respondent in Panel 2 who completed all 8 assessments would increase their odds of winning by earning 8 entries. The sweepstakes were conducted as a random drawing after the final assessment for each panel concluded. No empirical tests were conducted to measure the impact of incentivization.

### Engagement Strategies

As the study progressed, we implemented incremental ways to encourage participation. Texting on mobile devices is the most prevalent means of communication for Americans under 50 years of age [13]. To leverage this behavior, we introduced the option to have text reminders sent to mobile devices as an additional prompt to complete an assessment.

To help participants connect with the study and foster a sense of community, selected pregnancy and lifestyle top-line results were shared periodically with participants in assessment invitations. Results shared included the number of pregnant women actively participating in the study and facts about common pregnancy concerns and behaviors. At the study’s end, selected findings were also shared in an article hosted on the BabyCenter website, as participants had told us via feedback survey that they were interested to see what we had learned [14].

We closely monitored participation behaviors to identify chronic nonresponders, defined as participants that did not respond to two or more consecutive assessments. At four strategic intervals over the course of the study, before the more in-depth, longer *full* assessments were scheduled to deploy, dedicated emails were sent specifically to nonresponders in addition to the standard invitation protocol, asking them to return to active participation and reminding them of the potential to earn new entries into the sweepstakes.

## Results

### Recruitment

In 26 nonconsecutive calendar days, 476,863 invitation impressions were served, garnering 5843 clicks (1.2% click rate). This rate was typical for the floater intercept recruitment methodology used by BabyCenter as per their internal data. Industry benchmarks for random intercept survey invitations are not readily available, but as proxy, the click rate on a typical website display ad unit in the health category was 0.31% [15]. A 2016 study with a niche user population utilizing Twitter as a recruitment source noted click rates between 0.43% and 0.50% on its targeted study recruitment ads [16].

We manipulated recruitment tactics to achieve a more representative profile of pregnant women. Those recruited on the weekend were more likely to be employed than those recruited during the week. Those recruited with targeting on desktop devices were more likely to be in older age groups, compared to those recruited via mobile devices. We tested the impact of inclusion and exclusion of the monetary incentive during intercept recruitment on the proportions of household income and determined that not mentioning the incentive increased participation among higher-income groups, but skewed the recruitment toward older women with a higher level of education attainment (see Table 2). The sampling approach was fine-tuned based on these learnings to yield the initial baseline sample.

**Table 2:** Results of selected recruitment tactics.

Participant characteristics^a^		Total participants					Participants where no incentive was offered			
		Recruited on a weekday, n (%)	*P* value	Recruited on a weekend, n (%)	*P* value	Recruited on a weekday, n (%)		*P* value	Recruited on a weekend, n (%)	*P* value
**Age (years)**										
	Total	371 (100)		389 (100)		135 (100)			43 (100)	
	18-24	98 (26.5)	.06	82 (21.1)	.19	33 (24.4)		.72	5 (11.6)	.06
	25-34	208 (56.9)	.36	237 (60.9)	.11	68 (50.4)		.06	30 (69.8)	.11
	≥35	65 (17.5)	.39	70 (18.0)	.56	34 (25.2)		.04	8 (18.6)	.96
**Household income (US $)**										
	Total	338 (100)		353 (100)		124 (100)			40 (100)	
	<25,000	101 (29.9)	.11	91 (25.8)	.54	30 (24.2)		.46	8 (20.0)	.31
	25,000-49,999	89 (26.3)	.67	102 (28.9)	.33	31 (25.0)		.56	10 (25.0)	.76
	50,000-99,999	103 (30.5)	.52	98 (27.8)	.43	33 (26.6)		.49	16 (40.0)	.13
	≥100,000	45 (13.3)	.03	62 (17.6)	.58	30 (24.2)		.02	6 (15.0)	.76
**Employment status**										
	Total	371 (100)		385 (100)		134 (100)			43 (100)	
	Full time	142 (38.3)	<.001	200 (51.9)	.01	71 (53.0)		.11	22 (51.2)	.54
	Not employed full time	229 (61.7)	<.001	185 (48.1)	.01	63 (47.0)		.11	21 (48.8)	.54
**Educational level**										
	Total	368 (100)		388 (100)		132 (100)			45 (100)	
	High school or less	80 (21.7)	.81	81 (20.9)	.78	33 (25.0)		.27	5 (11.1)	.09
	Some college	130 (35.3)	.35	128 (33.0)	.76	34 (25.8)		.04	21 (46.7)	.06
	4-year degree or higher	158 (42.9)	.28	179 (46.1)	.60	65 (49.2)		.30	19 (42.2)	.69

^a^Excludes participants that preferred not to disclose their demographics.

Of the 5028 respondents who started the baseline assessment, 1557 completed it and met the inclusion criteria. The most common reasons for disqualification were pregnancy week out of target range, not pregnant, participating in other research, and out of target age range (see Table 3).

A total of 1179 participants met the eligibility requirements, completed the baseline screening survey, and opted to participate. While the panel recruited more quickly than we planned, the panel size was slightly shy of our target, as a few responses showed duplicate email addresses and were removed. This is a risk when using a digital recruitment method and offering gift card incentives. To mitigate this, we instituted email validation, which excluded baseline submissions from previously submitted email addresses, and monitored responses coming from the same IP addresses.

Two panels were recruited. Panel 1, with 858 women, was recruited early in the first trimester at weeks 4 to 10 of pregnancy. The 321 women in Panel 2, were recruited early in the third trimester at weeks 28 to 33 of pregnancy. Panel 2 was included in the event of undue attrition to insure a sufficient sample size in the critical postnatal period for future statistical modeling in health care research.

**Table 3:** Sample disposition.

Sample characteristics		Value, n (%)
Total site intercept impressions (n=476,863)		476,863 (100)
Clicks on site intercept survey, out of total impressions (n=476,863)		5843 (1.2)
Baseline assessment survey starts, out of total clicks (n=5843)		5028 (86.1)
**Disqualified participants, out of number of starts (n=5028)**		
	Total disqualified^a^	3471 (69.0)
	Pregnancy week not within targets	2186 (43.5)
	Did not complete the screening section	557 (11.1)
	Not pregnant	317 (6.3)
	Participating in other research	190 (3.8)
	Age outside range (ie, <18 years of age)	151 (3.0)
	Outside the United States	75 (1.5)
	Male	55 (1.1)
Qualified participants, out of number of starts (n=5028)		1557 (31.0)
Agreed to participate, out of qualified respondents (n=1557)		1535 (98.6)
Completed baseline survey^b^, out of respondents who agreed to participate (n=1535)		1179 (76.8)

^a^Respondents could have more than one disqualifier.

^b^Duplicate entries from the same email address were removed.

### Participation and Retention

Of the 1179 participants initially enrolled at baseline, 79.2% (934/1179) completed at least one additional assessment, 65.6% (773/1179) informed us about the birth of their child, 63.7% (751/1179) completed one or more assessments in the postpartum period, and 60.1% (709/1179) completed the final assessment in the study. There were 245 out of 1179 women enrolled in the study that did not return to take any additional assessments after baseline (20.8%) (see Table 4).

**Table 4:** Study attrition and retention into the postpartum period.

Attrition and retention groups		Value (N=1179), n (%)
Total participants enrolled at baseline		1179 (100)
**Participant attrition**		
	Total who dropped out	429 (36.4)
	Dropped out after baseline	245 (20.8)
	Dropped out after postpartum period	184 (15.6)
**Postpartum retention of participants**		
	Total retained	750 (63.6)
	Completed pregnancy and postpartum assessments	736 (62.4)
	Completed postpartum assessment only	14 (1.2)

A total of 45.1% (532/1179) of women completed all potential full surveys: 351 out of 532 (66.0%) in Panel 1 and 181 out of 532 (34.0%) in Panel 2. By the end of the study, 2.2% of participants (26/1179) actively opted out of the study, some noting pregnancy loss and others providing no reason.

Participation rates for each assessment varied and were impacted by the type of assessment, the incentives offered, and the position in the protocol. Short assessments and long assessments showed similar cooperation rates—64.6% (4669/7222) and 65.0% (3088/4754), respectively—but attributing cooperation to survey length alone cannot be established, as we put more effort into garnering responses to longer surveys.

After closing recruitment for the fifth assessment after baseline (ie, time point [T] 6 [T6]) with a 51.6% (431/835) participation rate (see Table 5), we began aggressively implementing re-engagement strategies starting with the next full survey at T7. Strategies included revising email invitation copy, sending dedicated correspondence to nonresponders, and implementing text reminders.

Completion rate trends point to engagement strategies boosting the total number of assessment surveys completed. Following T6, which had a cooperation rate of 51.6% (431/835), cooperation began to increase, with cooperation rates of 58.5% (490/837) at T7, 59.9% (692/1156) at T8, 59.8% (499/835) at T9, and 64.2% (742/1156) at T10. Among the 370 participants that opted in for text reminders, response rates improved by as much as 40% over the group that did not opt in. Communications sent to nonresponders during pregnancy encouraged 229 nonengaged participants to re-engage with the study and complete future assessments. A portion of these nonresponders may have returned on their own without re-engagement efforts; however, that proportion is unknown.

The attrition of participants after giving birth was expected, as this pivotal event shifts priorities. We were pleased to retain 80.4% (751/934) of the active sample after this life-changing point in time. In fact, the T12 assessment was administered 0 to 5 days after giving birth and achieved a 93.4% (465/498) participation rate. This reaffirmed our confidence in the approach and ability to continue measurement of the pregnancy sample into the postnatal period.

**Table 5:** Participation rate by assessment instrument and time point.

Time point (T)		Assessment instrument	Invitations, n (%)^a^	Completed assessments out of number of invitations, n (%)
**Pregnancy (n=858)**				
	T1 (Panel 1: weeks 4-10; Panel 2: weeks 29-33) (n=1179)	Baseline assessment	476,863^b^	1179^b^
	T2 (Panel 1: weeks 6-11)	Mini A^c^	853 (99.4)	538 (63.1)
	T3 (Panel 1: weeks 9-11)	Mini B^d^	853 (99.4)	469 (55.0)
	T4 (Panel 1: weeks 12 and 13)	Full^e^	840 (97.9)	482 (57.4)
	T5 (Panel 1: week 15)	Mini A	835 (97.3)	448 (53.7)
	T6 (Panel 1: week 18)	Mini B	835 (97.3)	431 (51.6)
	T7 (Panel 1: week 21)	Full	837 (97.6)	490 (58.5)
	T8 (Panel 1: week 25; Panel 2: week 32) (n=1179)	Mini A	1156 (98.0)	692 (59.9)
	T9 (Panel 1: week 28)	Mini B	835 (99.4)	499 (59.8)
	T10 (Panel 1: week 32; Panel 2: week 35) (n=1179)	Full	1156 (98.0)	742 (64.2)
	T11 (weeks 38-42) (n=1179)	Birth survey	1156 (98.0)	773 (66.9)
**Postpartum (n=773)^f^**				
	T12 (+2 days)	Mini B	498 (64.4)	465 (93.4)
	T13 (week 1)	Mini A	594 (76.8)	539 (90.7)
	T14 (week 4)	Full	768 (99.3)	665 (86.6)
	T15 (week 8)	Mini B	763 (98.7)	588 (77.1)
	T16 (week 12) (n=1179)	Final assessment^g^	1153 (97.8)	709 (61.5)

^a^The number of invitations for each assessment varied due to women opting out and opting back in as the study progressed.

^b^Recruitment at baseline was performed via random intercept, versus email invitations as with subsequent assessments; 476,863 represents the number of site impressions for the intercept and 1179 represents total participants enrolled at baseline.

^c^The Mini A (short-form) instrument contained five psychometric questions.

^d^The Mini B (short-form) instrument contained four psychometric questions.

^e^The Full (long-form) survey contained six psychometric questions.

^f^In the postpartum period, the length of time that had elapsed from giving birth to responding to the birth survey determined which assessment a respondent was next eligible to complete, which also impacted the number of invitations sent. The invitations sent during the postpartum period were only sent to those women who had confirmed the birth of her child via the birth survey.

^g^All respondents, regardless of birth survey response, were invited to take the final assessment.

Two population-based maternity studies with similar assessment timing allowed for a remedial comparison of participation statistics: the MARI Study, a longitudinal study conducted among pregnant women recruited from community clinics in Dresden, Germany, and the GUSTO Study, which was conducted among families in Singapore recruited during their first clinical visit of pregnancy and then followed through birth and 36 months postpartum [6,7]. In the late–second trimester and early–third trimester assessments, in which the EPDS or similar instruments were administered, the BabyCenter study had a participation rate (529/858, 61.7%) that was within the range of the MARI Study (57.6%) and the GUSTO Study (77.5%). For assessments conducted at approximately 3 to 4 months postpartum, all three studies showed remarkably similar participation rates, ranging from 57.7% (719/1247) for the GUSTO Study to 59.3% (509/858) for the BabyCenter study (see Table 6).

**Table 6:** Comparison of participation rates in longitudinal perinatal depression studies.

Participant details at each time point		BabyCenter longitudinal study of perinatal mood disorders (United States) (n=858)^a^	MARI^b^ Study (Germany) [6] (n=483)	GUSTO^c^ Study (Singapore) (n=1247) [7]
**Qualified at baseline**				
	Pregnancy weeks	4-10	10-12	<14
	Participants, n (%)	858 (100)	483 (100)	1247 (100)
**Pregnancy assessment**				
	Pregnancy weeks	32	35-37	26
	Participants, n (%)	529 (61.7)	278 (57.6)	967 (77.5)
**Postpartum assessment**				
	Postpartum months	3 months	4 months	3 months
	Participants, n (%)	509 (59.3)	283 (58.6)	719 (57.7)

^a^Only Panel 1 participants were included.

^b^MARI: Maternal Anxiety in Relation to Infant Development.

^c^GUSTO: Growing Up in Singapore Towards healthy Outcomes.

### Population Profile

At baseline, the profile of participants was similar to the population of women and births in the United States for age, marital status, presence of children, employment, and ethnicity [9,17]. The study sample had a higher concentration of women who had achieved a college or higher education degree, consistent with an online population [18]. Participants in the study demonstrated lower median household income than the US median [19]. This is potentially a result of the monetary incentives offered.

Attrition that occurred over the course of the study period is not inconsequential for demographic characteristics, with potential impact on mood-related characteristics as well. Participants retained through completion of the final assessment demonstrated a sample profile that differed from the baseline profile. The sample at final assessment showed higher median age, higher household income, higher incidence of marriage, and higher education attainment. This subset also demonstrated a different ethnic makeup, with a higher proportion reporting ethnicity as White, and fewer identifying as African American, Black, or Hispanic (see Table 7). Attrition characteristics are similar to those from other perinatal studies, such as the EDEN study (Etude sur les déterminants pré et post natals précoces du Développement psychomoteur et de la santé de l’ENfant), the mother-child EDEN cohort study based in France [20].

Participants completing the final assessment showed similar characteristics for number of babies, type of birth, and birth week.

Table 8 shows the birthing profile of participants determined during the final assessment.

**Table 7:** Participant profile ascertained at baseline and at the final assessment versus US births.

Participant characteristics			Baseline respondents: 4-10 weeks pregnant (N=1179), n (%)		Final respondents: 12 weeks postpartum (n=709), n (%)		US births (n=3,945,875), n (%)	
Have two or more children, including current pregnancy			697 (59.1)		419 (59.1)		2,445,998 (62.0) [9]	
Marital status: married			699 (59.3)		815 (68.1)		2,376,079 (60.2) [9]	
Employment status: employed			759 (64.4)		748 (62.5)		2,493,453/3,939,144 (63.3) [17]	
Education: 4-year college degree or higher			561 (47.6)		652 (54.5)		1,262,680 (32.0) [9]	
**Single race**								
	White	656 (55.6)		701 (58.6)		2,056,332 (52.1) [9]		
	Black or African American	178 (15.1)		142 (11.9)		558,622 (14.2) [9]		
	Asian or Pacific Islander	53 (4.5)		68 (5.7)		254,471 (6.4) [9]		
Ethnicity: Hispanic (any)			225 (19.1)		186 (15.5)		918,447 (23.3) [9]	
**Age of mother in years**								
	15-24^a^	254 (21.5)		123 (17.3)		1,013,787 (25.7) [9]		
	25-29	344 (29.2)		211 (29.8)		1,149,122 (29.1) [9]		
	30-34	359 (30.4)		223 (31.5)		1,111,042 (28.2) [9]		
	35-39	183 (15.5)		130 (18.3)		547,488 (13.9) [9]		
	40-44	40 (3.4)		22 (3.1)		113,140 (2.9) [9]		
**Annual household income (US $) (US births n=** **3,969,962)**								
	<25,000	199 (23.2)		131 (18.5)		640,062 (16.1) [19]		
	25,000-49,999	211 (24.6)		171 (24.1)		828,406 (20.9) [19]		
	50,000-74,999	123 (14.3)		117 (16.5)		705,117 (17.8) [19]		
	75,000-99,999	98 (11.4)		93 (13.1)		559,027 (14.1) [19]		
	≥100,000	154 (17.9)		142 (20.1)		1,237,350 (31.2) [19]		
	Prefer not to answer	73 (8.5)		55 (7.8)		N/A^b^		

^a^The National Center for Health Statistics (NCHS) reports births by the following age ranges of the mother: *Under 15*, *15-19*, and *20-24 years;* the BabyCenter study reports births by the mother’s age starting at 18 years.

^b^N/A: not applicable. The survey instruments in this study permitted respondents to opt out of providing personal information by selecting *Prefer not to answer*. NCHS reports characteristics for the entire population.

**Table 8:** Birthing profile ascertained in final assessment.

Participants’ birthing details		Final respondents: 12 weeks postpartum (n=709), n (%)	US births [9] (n=3,945,875), n (%)
**Birth location**			
	Hospital	667 (94.1)	3,883,255 (98.4)
	Birthing center	30 (4.2)	19,767 (0.5)
	At home	7 (1.0)	38,830 (1.0)
**Number of babies**			
	Single	694 (97.9)	3,810,149 (96.6)
	Twins or multiples	15 (2.1)	135,726 (3.4)
**Type of birth**			
	Vaginal	496 (70.0)	2,684,803 (68.0)
	Caesarean section	213 (30.0)	1,258,581 (31.9)
**Birth term**			
	Full (≥39 weeks)	467 (65.9)	2,551,797 (64.7)
	Early (37 or 38 weeks)	172 (24.3)	1,005,014 (25.5)
	Preterm (≤36 weeks)	70 (9.8)	388,669 (9.9)

### Data Set Validation

We investigated the factor structure of the psychometric scales and compared these to previously published results. The EPDS measurement of Panel 1 at baseline, despite exclusion of the suicidality item, was similar in structure to published results from the Postpartum Depression: Action Towards Causes and Treatment (PACT) Consortium, with three analogous factors of mood disorder: depressed mood, anxiety, and anhedonia (see Table 9) [21]. The Obsessive-Compulsive Inventory was noted to be remarkably similar in structure to the published version (see Multimedia Appendix 3) [22].

**Table 9:** Factor structure of the Edinburgh Postnatal Depression Scale (EPDS) and comparison with the Postpartum Depression: Action Towards Causes and Treatment (PACT) study.

EPDS item (item No.)	PACT: relative contributions of EPDS items to dimensions and factors [21], factor score				BabyCenter EPDS factor analysis at baseline: Panel 1 (n=858), factor score		
	Depressed mood	Anxiety	Anhedonia	Depressed mood		Anxiety	Anhedonia
Suicidal thoughts (10)	97	–17	–2	N/A^a^		N/A^a^	N/A^a^
Unhappy: crying (9)	79	19	4	80		1	5
Unhappy: difficulty sleeping (7)	76	15	4	66		7	6
Felt scared or panicky (5)	51	41	0	6		71	4
Felt sad or miserable (8)	51	44	–2	74		7	11
Anxious or worried (4)	3	74	1	–5		75	12
Things on top of me, difficulty coping (6)	11	68	–7	41		26	17
Looked forward with enjoyment (2)	–2	2	83	9		–3	81
Been able to laugh (1)	–7	8	81	6		3	78
Blamed myself unnecessarily (3)	13	–17	57	18		56	–14

^a^This item and dimension was not included in EPDS instrument in the BabyCenter Study.

### Participant Feedback

After completing the final assessment, we offered participants the opportunity to provide feedback about their overall experience via a survey. Overall, 61.0% of participants active in the postpartum period (459/752) provided feedback.

Of those who responded to this feedback survey, 98.3% (451/459) were *satisfied* or *very satisfied* with their experience participating in the study, 86.7% (398/459) felt the incentives were *very fair*, 91.5% (420/459) said the number of questions in each survey was *the right amount*, and 89.5% (411/459) said the number of emails received in relation to the study was *the right amount*. We note that nonresponse bias in this assessment may not be inconsequential, as nonresponders to the feedback survey were less engaged with the study; overall, they completed 18% fewer assessments than responders in the postpartum period.

## Discussion

### Overview

In this paper, we showed that it is possible to recruit a large and representative sample of pregnant women into an online panel via the BabyCenter website. We implemented a range of methods to keep participants active and reduce attrition. Our panel provided high-quality data that can now be used to learn new insights into mental health during and shortly after pregnancy.

### Lessons Learned

In this study we demonstrated that leveraging digital methods to measure a niche population over a length of time to collect a longitudinal data set is both viable and logical, as digital methods afford the following:

1. Ability to reach a specific population with a digital media partner.
2. Capability to recruit a large convenience sample into an online panel in a short period of time.
3. Capacity to readily adjust recruitment strategies to help construct a more representative panel profile.
4. Tools to automate and optimize otherwise tedious processes when collecting repeated measures (ie, API).
5. Flexibility to easily introduce additional retention elements as needed.
6. Means to execute longitudinal data collection for the validation of existing knowledge and the advancement of scientific study.

We were able to recruit a large and representative sample of pregnant women into an online panel during a 26-day period. The key recruitment lessons learned were as follows:

1. Partner with a website that is known to interact with the required population.
2. Adapt the demographic sampling parameters to get a representative population.
3. Use friendly language in the advert’s invitation copy that focuses on altruism.
4. Employ email or IP and time stamp validation to reduce duplicate and invalid participants.
5. Offer an initial incentive at enrollment that is fair but not overly generous to encourage legitimate enrollment.

The study duration was as long as 9 to 11 months from early pregnancy. Our online panel captured a baseline survey and one follow-up survey for approximately 80% of respondents and had similar attrition to previous longitudinal panel studies. The methods we used to reduce attrition were as follows:

1. Being transparent by providing details and expectations of the survey at enrollment so participants would know the required commitment.
2. Reducing monotony by alternating survey questions and varying survey lengths.
3. Adding friendly questions at the beginning of the survey about the participants’ experience to increase engagement.
4. Making the surveys easy to complete by optimizing them based on device (ie, desktop vs mobile devices).
5. Providing participants with interaction options (ie, text and email), but being careful not to unnecessarily overburden.
6. Sending personalized emails to chronic nonresponders and reminders of incentive status.
7. Using a combination of monetary and nonmonetary incentives, such as sharing study findings.

### Limitations

During the recruitment period, although the study invitations served on BabyCenter were randomized, there is no way to determine the characteristics of site visitors that chose not to click on the invitation. This is due to the anonymity of intercepting in a digital environment and online data privacy issues. To address this limitation, extra care was taken to monitor the composition and characteristics of the panel at all stages.

When using a digital-only methodology without the human-to-human contact that is often part of a clinical study approach with pregnant women, attrition is likely to be problematic. Of the participants who did not complete an additional assessment after baseline, attrition occurred disproportionally within Panel 1. Recruitment of Panel 1 participants occurred very early in pregnancy, at 4 to 10 weeks, when rates of pregnancy loss and false positives can be as high as 20%. Although we did receive participant-initiated requests to opt out, it is likely that a portion of women who experienced pregnancy loss or false positives did not notify us and did not return to complete another assessment. We had no alternative means to contact these women.

It is also realistic to assume that the incentive for completing the baseline assessment, a US $25 e-gift card, was sufficient reward for some women who chose not to continue in the study. We hypothesize that a smaller reward at enrollment may have extended the period needed to recruit the target number of participants but resulted in higher cooperation rates.

As stated, the study design did not include direct contact between participants and researchers, unless an inquiry was initiated by the participant. This was intentional but created another limitation. We chose not to include the suicidality item in the EPDS scale, confining the measurement and analysis to only 9 of the 10 standard items. Without the appropriate means to support women that may have expressed an inclination toward self-harm, we chose to exclude it. We provided links to suicide prevention and mental health resources in the study materials. We do not believe the omission of suicidality measurement has hampered achievement of the overall study objective but does create an unknowable gap in the data set.

Digital surveys may offer the advantage of increased accuracy with the convenience and anonymity they afford. Results from one perinatal depression study demonstrated that responses submitted by mail showed higher EPDS scores compared to responses collected by phone [23]. Another investigation found that women preferred to complete the EPDS assessment in the more comfortable environment of their own home versus in a clinical setting, in which interacting with a researcher impacted how women responded [24]. Testing this hypothesis was not within the scope of our study.

There are challenges to contextualizing results with other studies. To our knowledge, longitudinal studies from pregnancy to the postpartum period conducted exclusively online have not been published. Comparing a perinatal sample to population studies of different nonmaternal targets is problematic due to the nature of the birth of a child, a pivotal component of attrition. It is difficult to compare the participation rates of this study to prior perinatal depression research due to the inclusion in our study of women early in pregnancy at 4 to 10 weeks of gestation, and the fact that many other studies were conducted with patients recruited later in their pregnancies in clinical settings. That said, two other population-based longitudinal studies of perinatal depression with similar assessment time frames showed comparable retention rates at about 3 to 4 months postpartum.

### Conclusions

Recruiting participants into an online panel from a trusted digital media source and administering a well-designed study exclusively in an online environment can successfully be utilized for scientific research. We approached this study with a focus on maximizing engagement, reducing attrition, and building trust with participants, which resulted, to the best of our knowledge at the time, in the collection of the largest, most comprehensive longitudinal data set to date measuring perinatal mood disorders from early pregnancy.

## Appendix

Multimedia Appendix 1 Assessment instruments.

Multimedia Appendix 2 General interest and health questions.

Multimedia Appendix 3 Obsessive-Compulsive Inventory factor structure.

## Abbreviations

API: application programming interface
EDEN: Etude sur les déterminants pré et post natals précoces du Développement psychomoteur et de la santé de l’ENfant
EPDS: Edinburgh Postnatal Depression Scale
GUSTO: Growing Up in Singapore Towards healthy Outcomes
MARI: Maternal Anxiety in Relation to Infant Development
PACT: Postpartum Depression: Action Towards Causes and Treatment
T: time point
