# Supplementary material for: Leveraging Digital Technology in Conducting Longitudinal Research on Mental Health in Pregnancy: Longitudinal Panel Survey Study
Source: JMIR Pediatr Parent. 2021 Apr 27;4(2):e16280. doi: 10.2196/16280 (PMC8114159; doi:10.2196/16280)
Supplement: Multimedia Appendix 1 [file pediatrics_v4i2e16280_app1.docx]

**Lessons Learned: Leveraging Digital Technology in Conducting Longitudinal Research on Mental Health in Pregnancy: Longitudinal, Panel Survey Study**

**Multimedia Appendix 1**

**Assessment Instruments**

**Baseline Assessment Questionnaire**

**Q1 Thank you for agreeing to complete this survey. Please answer these next few questions to see if you qualify to participate in our research study. Do you live in the United States?**

Yes

No

**Q2 What is your gender?**

Male

Female

**Q3 How old are you?**

17 or younger

18-24

25-29

30-34

35-39

40-44

45 or older

**Q4 Are you currently pregnant?**

Yes

No

**Q5 Are you currently participating in any ongoing clinical or medical health studies?**

Yes

No

*Answer If Are you currently pregnant? Yes Is Selected*

**Q6 Congratulations! How far along are you in your pregnancy?**

1 week

2 weeks

3 weeks

4 weeks

5 weeks

6 weeks

7 weeks

8 weeks

9 weeks

10 weeks

11 weeks

12 weeks

13 weeks

14 weeks

15 weeks

16 weeks

17 weeks

18 weeks

19 weeks

20 weeks

21 weeks

22 weeks

23 weeks

24 weeks

25 weeks

26 weeks

27 weeks

28 weeks

29 weeks

30 weeks

31 weeks

32 weeks

33 weeks

34 weeks

35 weeks

36 weeks

37 weeks

38 weeks

39 weeks

40 weeks

41 weeks

42 weeks

**Q7 What is your expected due date?**

Month

Day

Year

**Q8 Good news! You qualify to participate in our survey. This survey is part of a larger research study being conducted by BabyCenter to help understand women’s health and well-being during pregnancy and in the first few months after their baby’s birth. Our survey today will take less than 10 minutes to complete and at the end of it you will receive a $25 Amazon gift certificate if you agree to participate in our larger study.**

*Answer If cohort Is Equal to 1*

**Q9 Here is more info on our research study: You’ll receive a number of surveys to complete over the rest of your pregnancy and your baby’s first 3 months. Most surveys will take less than 5 minutes to complete, a few may take up to 10 minutes. For each survey you complete you’ll receive points to redeem for Amazon gift certificates. You can earn as much as $180 for completing all surveys! What’s more, after the final survey in the study is completed, there will be a random drawing for a $1,000 cash grand prize – every survey you complete will earn you an entry into the drawing, so the more surveys you complete, the greater your chances of winning. And you’ll be helping us with some important research that will benefit future pregnancies and babies.**

Answer If cohort Is Equal to 2

**Q10 Here is more info on our research study: You’ll receive 7 short surveys to complete over the rest of your pregnancy and your baby’s first 3 months. Most surveys will take less than 5 minutes to complete, a few may take up to 10 minutes. For each survey you complete you’ll receive points to redeem for Amazon gift certificates. You can earn as much as $125 for completing all surveys! What’s more, after the final survey in the study is completed, there will be a random drawing for a $1,000 cash grand prize – every survey you complete will earn you an entry into the drawing. The more surveys you complete, the greater your chances are of winning. And you’ll be helping us with some important research that will benefit future pregnancies and babies.**

**Q11 The data collected as part of this study will be used strictly for scientific research and education and will be used in accordance with BabyCenter’s** [**Privacy Policy**](https://www.babycenter.com/help-privacy)**. All data will be collectively analyzed, and your responses will remain strictly confidential. If you would like any further information about this research and participants rights, please contact us by email maternal.insights@babycenter.com.**

**Q12 If you agree to participate in this research study, please click on ‘I agree’.**

I agree

I don't agree

**Q13 Thank you for agreeing to participate! Your input in this important research study will benefit future pregnancies and babies. Let’s get started right away.**

**Q14 To begin, which statement best describes your reaction when you first found out you were** pregnant?

I was thrilled

I was happy

I was relieved

I was angry

I was sad

I had mixed emotions

**Q15 How have you been feeling so far in this pregnancy?**

Great

Pretty good

Pretty bad

Terrible

**Q16 Have you experienced any of the following during this pregnancy? (Please select all that apply.)**

Nausea

Difficulty eating (e.g., motivation to eat healthy, appetite)

*If cohort Is Equal to 2*

Difficulty managing your weight

Fatigue or lack of energy

Back pain

*If cohort Is Equal to 2*

Gestational diabetes

Headaches or migraines

Insomnia or trouble sleeping

Mood swings

Anxiety

Depression

*If cohort Is Equal to 2*

Preeclampsia

High blood pressure

Other, please specify: ____________________

None of these

**Q17 Have you ever been pregnant before?**

Yes

No

*Answer If Have you ever been pregnant before? Yes Is Selected*

**Q18 Did you experience any of the following in previous pregnancies? (Please select all that apply.)**

Nausea

Difficulty eating (e.g., motivation to eat healthy, appetite)

Difficulty managing your weight

Fatigue or lack of energy

Back pain

Headaches or migraines

Insomnia or trouble sleeping

Mood swings

Anxiety

Depression

Gestational diabetes

High blood pressure

Preeclampsia

Miscarriage

Pre-term birth

Stillbirth

Delivery complications

Baby placed in NICU

Other, please specify: ____________________ ____________________

None of these

**Q19 Have you seen an obstetrician or other health care provider for prenatal care for this pregnancy?**

Yes, I have regularly scheduled appointments for prenatal care

Yes, I have had a prenatal checkup, but don’t plan for regular appointments

No, it’s too early in my pregnancy, but I do have a future appointment

No, but I plan to make a future appointment

No, I have no plans to seek prenatal care from a health care provider

*Answer If cohort Is Equal to 2*

**Q20 How would you describe your weight gain during this pregnancy?**

I am gaining less than the recommended amount

I am gaining the recommended amount

I am gaining more than the recommended amount

I am not sure

**Q21 Next we’ll be asking you some questions about how you’ve been feeling during this pregnancy. There are no right or wrong answers, as every woman responds differently to being pregnant. Please answer the questions as honestly as possible.**

**Q22 For each of the statements below please pick the response that best describes your present feeling. Do no spend too much time on any one statement.**

Not at all Somewhat Moderately Very much

I feel calm

I am tense

I feel upset

I am relaxed

I feel content

I am worried

**Q23 How healthy do you eat now that you’re pregnant?**

Extremely healthy

Very healthy

Pretty healthy

Not very healthy

Not at all healthy

*Answer If cohort Is Equal to 1*

**Q24 Which of these benefits of pregnancy are you most looking forward to? (Please select all that apply.)**

Becoming more health-conscious

Others holding doors open or giving up seats for me

Extra lush hair and strong nails

No need for birth control

Eating “what I want”

Extra cleavage

Buying new clothes/shoes

None of these

*Answer If cohort Is Equal to 2*

**Q25 Which of these have been surprising or enjoyable benefits of being pregnant? (Please select all that apply.)**

Becoming more health-conscious

Others holding doors open or giving up seats for me

Extra lush hair and strong nails

No need for birth control

Eating “what I want”

Extra cleavage

Buying new clothes/shoes

None of these

**Q26 Below are statements that describe emotions women may experience during pregnancy. Please read each one and select the answer that most closely matches how you have felt in the past 7 days:**

**Q27 I have been able to laugh and see the funny side of things.**

As much as I always could

Not quite so much now

Definitely not so much now

Not at all

**Q28 I have looked forward with enjoyment to things.**

As much as I ever did

Rather less than I used to

Definitely less than I used to

Hardly at all

**Q29 I have blamed myself unnecessarily when things went wrong.**

Yes, most of the time

Yes, some of the time

Not very often

No, never

**Q30 I have been anxious or worried for no good reason.**

No not at all

Hardly ever

Yes, sometimes

Yes, very often

**Q31 I have felt scared or panicky for no very good reason.**

Yes, quite a lot

Yes, sometimes

No, not much

No, not at all

**Q32 Things have been getting on top of me.**

Yes, most of the time I haven’t been able to cope at all

Yes, sometimes I haven’t been coping as well as usual

No, most of the time I have coped quite well

No, I have been coping as well as ever

**Q33 I have been so unhappy that I have had difficulty sleeping.**

Yes, most of the time

Yes, sometimes

Not very often

No, not at all

**Q34 I have felt sad or miserable.**

Yes, most of the time

Yes, sometimes

Not very often

No, not at all

**Q35 I have been so unhappy that I have been crying.**

Yes, most of the time

Yes, quite often

Only occasionally

No, never

**Q36 How bad is “morning sickness” for you during this pregnancy?**

Terrible

Pretty bad

Not too bad

I’m finally over it thankfully!

Never had any

**Q37 Here are some statements that describe how women may respond to everyday life during pregnancy. How often in the last month have you felt each of the following?**

Never Almost never Sometimes Fairly often Very often

That you were unable to control the important things in your life

Confident about your ability to handle your personal problems

That things were going your way

Difficulties were piling up so high that you could not overcome them

**Q38 Now we’d like to know a bit more about your family and social situation, and more specifically, how supported you feel in your pregnancy.**

**Q39 How often have you felt the sentiment stated in each of the sentences below?**

Never Rarely Sometimes Usually Always

I have someone who will listen to me when I need to talk

I have someone to confide in or talk to about myself or my problems

I have someone who makes me feel appreciated

I have someone to talk with when I have a bad day

**Q40 Thinking about how you’ve been feeling more recently, over the last 2 weeks, how often have you been bothered by the following problems?**

Nearly every day Over half the days Several days Not at all

Feeling nervous, anxious, or on edge

Not being able to stop or control worrying

Worrying too much about different things

Trouble relaxing

Being so restless that it's hard to sit still

Becoming easily annoyed or irritable

Feeling afraid as if something awful might happen

Feeling I might lose control of myself

**Q41 Thank you so much for your answers! Almost done. But before you leave us today, we’d like to ask you a few, important questions about your health prior to your pregnancy.**

**Q42 How would you describe your general overall health prior to becoming pregnant?**

Excellent

Very Good

Good

Fair

Poor

**Q43 How would you describe your weight before you became pregnant?**

Extremely underweight

Moderately underweight

Slightly underweight

Normal weight

Slightly overweight

Moderated overweight

Extremely overweight

**Q44 How frequently did you do the following prior to becoming pregnant?**

Every day A few times a week Once a week Less often Never

Drink alcohol

Practice meditation or breathing

Smoke cigarettes

Exercise

Smoke marijuana

Eat healthy

Write in a journal or blog

Use illegal drugs

Practice yoga

Read a book

Participate in an online discussion group

Meet with friends

Eat junk food

Go for walks

*Answer If How frequently did you do the following prior to becoming pregnant? Smoke cigarettes - Every day Is Selected Or How frequently did you do the following prior to becoming pregnant? Smoke cigarettes - A few times a week Is Selected Or How frequently did you do the following prior to becoming pregnant? Smoke cigarettes - Once a week Is Selected Or How frequently did you do the following prior to becoming pregnant? Smoke cigarettes - Less often Is Selected*

**Q45 Now that you're pregnant, do you smoke cigarettes?**

Yes, but I'm thinking about quitting

Yes, and I don't plan to quit

No, I quit smoking when I started trying to conceive

No, I quit smoking when I found I out I was pregnant

No, I quit smoking a few weeks into my pregnancy

I prefer not to answer

**Q46 Has a medical professional ever diagnosed you with any of the following conditions? (Please select all that apply)**

Allergies

Diabetes (Type I or II)

Endometriosis

Fertility problems

Fibromyalgia

Hypertension (high blood pressure)

Irritable bowel syndrome (IBS)

Migraines

Obesity

Sleep disorders

Substance addiction

Other, please specify: ____________________

None of these

**Q47 Can you tell us, at any time prior to your pregnancy, had you suffered from any of the emotional or mental health conditions noted below? (Please select all that apply)**

Bipolar Disorder

Depression

Eating Disorder

Generalized Anxiety Disorder

Obsessive Compulsive Disorder (OCD)

Panic attacks

*If Have you ever been pregnant before? Yes Is Selected*

Postpartum Depression

Post-Traumatic Stress Disorder (PTSD)

Other emotional/mental health disorders

None of these

**Q48 Prior to this pregnancy, were you taking any of the following types of prescription medications for your mental health? (Please select all that apply.)**

Antidepressants

Anti-anxiety medications

Sleep medications

Other, please specify: ____________________

None of these

Prefer not to say

**Q49 Which of the following, if any, has happened to you within the past year? (Please select all that apply)**

Death in family or of a close friend

Divorced, separated or ended a relationship

Financial hardship

Legal problem/litigation

Had a miscarriage

Moved to a new location

Personally lost a job

Spouse/partner lost a job

Victim of a natural disaster

Victim of a non-violent crime (identity theft, burglary, etc.)

Victim of a violent crime

None of these

**Q50 For classification purposes only, please answer these final few questions about yourself.**

**Q51 Do you have any children?**

Yes

No

*Answer If Do you have any children? Yes Is Selected*

**Q52 What are the ages of your children? (Please select all that apply.)**

Less than 1 year old

1-2 years old

3-5 years old

6-12 years old

13-17 years old

18 years old or older

**Q53 What is your current marital status?**

Single

Married

Living with a partner

Divorced

Widowed

Other

**Q54 Which of the following best describes your current employment status?**

Employed and currently working full-time

Employed and currently working part-time

Unemployed

Stay at home mom

Student

Other

*Answer If What is your current marital status? Married Is Selected Or What is your current marital status? Living with a partner Is Selected*

**Q55 Does your spouse or partner suffer from any of the emotional or mental health conditions noted below? (Please select all that apply)**

Bipolar Disorder

Depression

Eating Disorder

Generalized Anxiety Disorder

Obsessive Compulsive Disorder (OCD)

Panic attacks

Post-Traumatic Stress Disorder (PTSD)

Other emotional/mental health disorders

None of these

**Q56 What is the highest level of education you have completed?**

Some high school

High school graduate

Some college

College graduate

Some post-graduate

Post graduate degree (e.g., Masters, PhD)

Prefer not to answer

**Q57 What is your ethnic background? (Please select all that apply)**

African American

Asian /Pacific Islander

Caucasian

Hispanic

Native American

Other

Prefer not to answer

**Q58 What is your annual household income before taxes?**

Under $25,000

$25,000 - $34,999

$35,000 - $49,999

$50,000 - $74,999

$75,000 - $99,999

$100,000 - $124,999

$125,000 or higher

Prefer not to answer

**Q59 Which US state to you currently live in?**

Alabama

Alaska

Arizona

Arkansas

California

Colorado

Connecticut

Delaware

District of Columbia

Florida

Georgia

Hawaii

Idaho

Illinois

Indiana

Iowa

Kansas

Kentucky

Louisiana

Maine

Maryland

Massachusetts

Michigan

Minnesota

Mississippi

Missouri

Montana

Nebraska

Nevada

New Hampshire

New Jersey

New Mexico

New York

North Carolina

North Dakota

Ohio

Oklahoma

Oregon

Pennsylvania

Rhode Island

South Carolina

South Dakota

Tennessee

Texas

Utah

Vermont

Virginia

Washington

West Virginia

Wisconsin

Wyoming

**Q60 Thank you for time today! Based on your responses, you qualify for a $25 Amazon gift card, and you could earn a lot more, for participating in our larger study.**

*Answer If cohort Is Equal to 1*

**Q61 More details about the study: You’ll be invited to complete a total of 14 surveys through the rest of your pregnancy and the first 3 months of your baby’s life. Most surveys will take only 5 minutes to complete. A few will be longer, up to 15 minutes. You could earn a total of $180 in Amazon gift cards if you complete all surveys. Surveys will be sent to you by email and you may complete them using your mobile phone or your computer, whichever is more convenient for you. You’ll earn points for completing each survey, and when you earn enough points, rewards will be sent directly to your email. You’ll still get points for completing new surveys even if you miss one or two. You’ll have a chance to win a $1,000 cash grand prize - every survey you complete earns you an entry into to the drawing, so the more surveys you complete, the greater your chances are to win! There are no obligations and you can opt out whenever you’d like.**

*Answer If cohort Is Equal to 2*

**Q62 More details about the study: You’ll be invited to complete a total of 7 surveys through the rest of your pregnancy and the first 3 months of your baby’s life. Most surveys will take only 5 minutes to complete. A few will be longer, up to 15 minutes. You could earn a total of $125 in Amazon gift cards if you complete all surveys. Surveys will be sent to you by email and you may complete them using your mobile phone or your computer, whichever is more convenient for you. You’ll earn points for completing each survey, and when you earn enough points, rewards will be sent directly to your email. You’ll still get points for completing new surveys even if you miss one or two. You’ll have a chance to win a $1,000 cash grand prize - every survey you complete earns you an entry into to the drawing, so the more surveys you complete, the greater your chances are to win! There are no obligations and you can opt out whenever you’d like.**

**Q63 In order to participate in the larger study and receive your first $25 Amazon gift card, please provide your first name and email address below. Your email address will only be used to invite you to complete new surveys, provide summaries of your points balance, and to send the Amazon gift cards you earn.**

First Name (Optional)

Email (Required)

**Q64 Please make sure to add maternal.insights@babycenter to your address book or list of "safe" senders so our emails don't get accidentally caught in your spam or junk folders**

**Q65 Thank you! We look forward to your participation. Please click next to have your first $25 Amazon gift card emailed to you right away!**

**Mini A Assessment Scales**

**Q1 Thank you for being part of this important study about women’s health that will help future moms and pregnancies. By completing today’s survey, you will earn $5 to your points total. When you reach $25 points, you may redeem them for a $25 Amazon gift card! Click the arrow to begin.**

**Q2 How much or how little your sleep met the following criteria in the past 7 days:**

Not at all A little bit Somewhat Quite a bit Very much

My sleep was refreshing

I had a problem with my sleep

I had difficulty falling asleep

**Q3 Sometimes sleep quality can have an impact on daily life. How much or how little have you felt each of the following situations in the past 7 days?**

Not at all A little bit Somewhat Quite a bit Very much

I had a hard time getting things done because I was sleepy

I felt alert when I woke up

I felt tired

I had problems during the day because of poor sleep

I had a hard time concentrating because of poor sleep

I felt irritable because of poor sleep

I was sleepy during the daytime

I had trouble staying awake during the day

**Q4 This next question is about physical discomfort you may or may not have experienced recently. Please respond to each question below about pain you may have felt in the past 7 days:**

Not at all A little bit Somewhat Quite a bit Very much

How much did pain interfere with your day to day activities?

How much did pain interfere with work around the home?

How much did pain interfere with your ability to participate in social activities?

How much did pain interfere with your household chores?

**Q5 Thank you for your responses thus far, your input is very helpful. Please answer these final questions about your overall state of mind as of late.**

**Q6 How frequently you have experienced each of the following feelings in the past 7 days:**

Never Rarely Sometimes Often Always

I felt fearful

I found it hard to focus on anything other than my anxiety

My worries overwhelmed me

I felt uneasy

**Q7 Over the past two weeks, how often have you been bothered by any of the following problems?**

Not at all Several days More than half the days Nearly every day

Little interest or pleasure in doing things

Feeling down, depressed, or hopeless

**Mini B Assessment Scales**

**Q1 Thank you for being part of this important study about women’s health that will help future moms and pregnancies. By completing today’s survey, you will earn $5 in points to your points total. When you reach $25 in points, you may redeem points for a $25 Amazon gift card! Your current point balance: ${m://Points}**

**Q2 You may recognize some of these next questions from a previous survey you have taken. As you progress in your pregnancy, your responses may change or they may stay the same. There are no right or wrong answers, and please be as honest as you can.**

**Q3 For each of the statements below please pick the response that best describes your present feeling. Do no spend too much time on any one statement.**

Not at all Somewhat Moderately Very much

I feel calm

I am tense

I feel upset

I am relaxed

I feel content

I am worried

**Q4 Below are statements that describe emotions women may experience during pregnancy. Please read each one and select the answer that most closely matches how you have felt in the past 7 days:**

**Q5 I have been able to laugh and see the funny side of things.**

As much as I always could

Not quite so much now

Definitely not so much now

Not at all

**Q6 I have looked forward with enjoyment to things.**

As much as I ever did

Rather less than I used to

Definitely less than I used to

Hardly at all

**Q7 I have blamed myself unnecessarily when things went wrong.**

Yes, most of the time

Yes, some of the time

Not very often

No, never

**Q8 I have been anxious or worried for no good reason.**

No not at all

Hardly ever

Yes, sometimes

Yes, very often

**Q9 I have felt scared or panicky for no very good reason.**

Yes, quite a lot

Yes, sometimes

No, not much

No, not at all

**Q10 Things have been getting on top of me.**

Yes, most of the time I haven’t been able to cope at all

Yes, sometimes I haven’t been coping as well as usual

No, most of the time I have coped quite well

No, I have been coping as well as ever

**Q11 I have been so unhappy that I have had difficulty sleeping.**

Yes, most of the time

Yes, sometimes

Not very often

No, not at all

**Q12 I have felt sad or miserable.**

Yes, most of the time

Yes, sometimes

Not very often

No, not at all

**Q13 I have been so unhappy that I have been crying.**

Yes, most of the time

Yes, quite often

Only occasionally

No, never

**Q14 These are a few statements that describe how women may respond to everyday life during pregnancy. How often in the last month have you felt each of the following?**

Never Almost never Sometimes Fairly often Very often

That you were unable to control the important things in your life

Confident about your ability to handle your personal problems

That things were going your way

Difficulties were piling up so high that you could not overcome them

**Q15 Now we’d like to know a bit more about your family and social situation, and more specifically, how supported you feel in your pregnancy.**

**Q16 How often have you felt the sentiment stated in each of the sentences below:**

Never Rarely Sometimes Usually Always

I have someone who will listen to me when I need to talk

I have someone to confide in or talk to about myself or my problems

I have someone who makes me feel appreciated

I have someone to talk with when I have a bad day

**Full Assessment Scales**

**Q1 Welcome back and thank you again for being part of this important study about women’s health that will help future moms and pregnancies. By completing today’s survey, you will add $15 in points to your total. When you reach $25 in points, you may redeem points for a $25 Amazon gift card! Your current point balance: ${m://Points}**

**Q2 You may recognize some of these next questions from a previous survey you have taken. As you progress in your pregnancy, your responses may change or they may stay the same. There are no right or wrong answers, and please be as honest as you can.**

**Q3 For each of the statements below please pick the response that best describes your present feeling. Do no spend too much time on any one statement.**

Not at all Somewhat Moderately Very much

I feel calm

I am tense

I feel upset

I am relaxed

I feel content

I am worried

**Q4 Below are statements that describe emotions women may experience during pregnancy. Please read each one and select the answer that most closely matches how you have felt in the past 7 days:**

**Q5 I have been able to laugh and see the funny side of things.**

As much as I always could

Not quite so much now

Definitely not so much now

Not at all

**Q6 I have looked forward with enjoyment to things.**

As much as I ever did

Rather less than I used to

Definitely less than I used to

Hardly at all

**Q7 I have blamed myself unnecessarily when things went wrong.**

Yes, most of the time

Yes, some of the time

Not very often

No, never

**Q8 I have been anxious or worried for no good reason.**

No not at all

Hardly ever

Yes, sometimes

Yes, very often

**Q9 I have felt scared or panicky for no very good reason.**

Yes, quite a lot

Yes, sometimes

No, not much

No, not at all

**Q10 Things have been getting on top of me.**

Yes, most of the time I haven’t been able to cope at all

Yes, sometimes I haven’t been coping as well as usual

No, most of the time I have coped quite well

No, I have been coping as well as ever

**Q11 I have been so unhappy that I have had difficulty sleeping.**

Yes, most of the time

Yes, sometimes

Not very often

No, not at all

**Q12 I have felt sad or miserable.**

Yes, most of the time

Yes, sometimes

Not very often

No, not at all

**Q13 I have been so unhappy that I have been crying.**

Yes, most of the time

Yes, quite often

Only occasionally

No, never

**Q14 These are a few statements that describe how women may respond to everyday life during pregnancy. How often in the last month have you felt each of the following?**

Never Almost never Sometimes Fairly often Very often

That you were unable to control the important things in your life

Confident about your ability to handle your personal problems

That things were going your way

Difficulties were piling up so high that you could not overcome them

**Q15 Now we’d like to know a bit more about your family and social situation, and more specifically, how supported you feel in your pregnancy.**

**Q16 How often have you felt the sentiment stated in each of the sentences below:**

Never Rarely Sometimes Usually Always

I have someone who will listen to me when I need to talk

I have someone to confide in or talk to about myself or my problems

I have someone who makes me feel appreciated

I have someone to talk with when I have a bad day

**Q17 Thinking about how you’ve been feeling more recently, over the last 2 weeks, how often have you been bothered by the following problems?**

Nearly every day Over half the days Several days Not at all

Feeling nervous, anxious, or on edge

Not being able to stop or control worrying

Worrying too much about different things

Trouble relaxing

Being so restless that it's hard to sit still

Becoming easily annoyed or irritable

Feeling afraid as if something awful might happen

Feeling I might lose control of myself

**Q18 Now we’d like to show you a few statements that describe experiences some pregnant women may have during the course of everyday life. Select the option that best describes how much or how little that experience has distressed or bothered you during the past month.**

Not at all A little Moderately A lot Extremely

I have saved up so many things that they get in the way.

I check things more often than necessary.

I get upset if objects are not arranged properly.

I feel compelled to count while I am doing things.

I find it difficult to touch an object when I know it has been touched by strangers or certain people.

I find it difficult to control my own thoughts.

I collect things I don’t need.

I repeatedly check doors, windows, drawers, etc.

I get upset if others change the way I have arranged things.

I feel I have to repeat certain numbers.

I sometimes have to wash or clean myself simply because I feel contaminated.

I am upset by unpleasant thoughts that come into my mind against my will.

I avoid throwing things away because I am afraid I might need them later.

I repeatedly check gas and water taps and light switches after turning them off.

I need things to be arranged in a particular order.

I feel that there are good and bad numbers.

I wash my hands more often and longer than necessary.

I frequently get nasty thoughts and have difficulty in getting rid of them.

**Final Assessment Questionnaire**

**Q1 Pregnancy is quite a journey, and we are so thankful that you have taken the time to share your experiences. The health of moms and babies is of utmost importance to us, and your responses have helped us to better understand pregnancy and motherhood. This is the final survey you will receive as a participant in this study. It should take 15 minutes or less to complete, and upon finishing you will receive $50 in Amazon gift cards to be awarded to you immediately in two $25 gift codes. Additionally, this is your last opportunity to earn an entry into the random drawing for a $1000 cash prize.**

**Q2 Let’s get started with the final survey!**

**Q3 To begin, we would like to learn about your recent birth experience. Please tell us, how would you describe your delivery?**

Very easy

Somewhat easy

Somewhat difficult

Very difficult

**Q4 Where did your delivery take place?**

Hospital

Birthing center

Other medical facility

At home

Other, please specify: ____________________

**Q5 Which of the following describes your birth?**

Singlet

Twins

Triplets+

**Q6 What type of delivery did you experience?**

Vaginal

C-section

**Q7 Did you deliver your baby pre-term (before 40 weeks)?**

Yes

No

**Q8 When did you deliver your baby?**

39 weeks

38 weeks

37 weeks

36 weeks

35 weeks

34 weeks

33 weeks

32 weeks

31 weeks

30 weeks

29 weeks

28 weeks

27 weeks

26 weeks or earlier

**Q9 Now, thinking back to your pregnancy, at any time did you experience any of the following? (Please select all that apply.)**

Nausea

Difficulty eating (e.g., motivation to eat healthy, appetite)

Difficulty managing your weight

Fatigue or lack of energy

Back pain

Gestational diabetes

Headaches or migraines

Insomnia or trouble sleeping

Mood swings

Anxiety

Depression

Preeclampsia

High blood pressure

Other, please specify: ____________________

None of these

**Q10 You may recognize some of these next questions from previous surveys you have taken. After giving birth, your responses may change, or they may stay the same. There are no right or wrong answers, and please be as honest as you can.**

**Q11 For each of the statements below please pick the response that best describes your present feeling. Do no spend too much time on any one statement.**

Not at all Somewhat Moderately Very much

I feel calm

I am tense

I feel upset

I am relaxed

I feel content

I am worried

**Q12 Below are statements that describe emotions moms may experience. Please read each one and select the answer that most closely matches how you have felt in the past 7 days:**

**Q13 I have been able to laugh and see the funny side of things.**

As much as I always could

Not quite so much now

Definitely not so much now

Not at all

**Q14 I have looked forward with enjoyment to things.**

As much as I ever did

Rather less than I used to

Definitely less than I used to

Hardly at all

**Q15 I have blamed myself unnecessarily when things went wrong.**

Yes, most of the time

Yes, some of the time

Not very often

No, never

**Q16 I have been anxious or worried for no good reason.**

No not at all

Hardly ever

Yes, sometimes

Yes, very often

**Q17 I have felt scared or panicky for no very good reason.**

Yes, quite a lot

Yes, sometimes

No, not much

No, not at all

**Q18 Things have been getting on top of me.**

Yes, most of the time I haven’t been able to cope at all

Yes, sometimes I haven’t been coping as well as usual

No, most of the time I have coped quite well

No, I have been coping as well as ever

**Q19 I have been so unhappy that I have had difficulty sleeping.**

Yes, most of the time

Yes, sometimes

Not very often

No, not at all

**Q20 I have felt sad or miserable.**

Yes, most of the time

Yes, sometimes

Not very often

No, not at all

**Q21 I have been so unhappy that I have been crying.**

Yes, most of the time

Yes, quite often

Only occasionally

No, never

**Q22 These are a few statements that describe how women may respond to everyday life. How often in the last month have you felt each of the following?**

Never Almost never Sometimes Fairly often Very often

That you were unable to control the important things in your life

Confident about your ability to handle your personal problems

That things were going your way

Difficulties were piling up so high that you could not overcome them

**Q23 Now we’d like to know a bit more about your family and social situation, and more specifically, how supported you feel at this point in time.**

**Q24 How often have you felt the sentiment stated in each of the sentences below:**

Never Rarely Sometimes Usually Always

I have someone who will listen to me when I need to talk

I have someone to confide in or talk to about myself or my problems

I have someone who makes me feel appreciated

I have someone to talk with when I have a bad day

**Q25 When you need information, guidance, and/or support related to taking care of your baby, which of the following do you rely on? (Please select all that apply)**

Spouse/partner

Your mother

Other family members

Friends you talk to in person (or via phone/text/IM/email)

Friends you connect with on social media (Facebook, Twitter)

Other moms online (BabyCenter Community, What To Expect Community)

Other moms you meet in person/ Local support groups

Medical professionals (doctors, nurses)

Midwife/doula

Religious groups

Email newsletters

Internet/websites

Mobile apps

Books

Magazines

TV programs

Radio

Other, please specify: ____________________

None of these

**Q26 Thinking about how you’ve been feeling more recently, over the last 2 weeks, how often have you been bothered by the following problems?**

Nearly every day Over half the days Several days Not at all

Feeling nervous, anxious, or on edge

Not being able to stop or control worrying

Worrying too much about different things

Trouble relaxing

Being so restless that it's hard to sit still

Becoming easily annoyed or irritable

Feeling afraid as if something awful might happen

Feeling I might lose control of myself

**Q27 Now we’d like to show you a few statements that describe experiences some moms may have during the course of everyday life. Select the option that best describes how much or how little that experience has distressed or bothered you during the past month.**

Not at all A little Moderately A lot Extremely

I have saved up so many things that they get in the way.

I check things more often than necessary.

I get upset if objects are not arranged properly.

I feel compelled to count while I am doing things.

I find it difficult to touch an object when I know it has been touched by strangers or certain people.

I find it difficult to control my own thoughts.

I collect things I don’t need.

I repeatedly check doors, windows, drawers, etc.

I get upset if others change the way I have arranged things.

I feel I have to repeat certain numbers.

I sometimes have to wash or clean myself simply because I feel contaminated.

I am upset by unpleasant thoughts that come into my mind against my will.

I avoid throwing things away because I am afraid I might need them later.

I repeatedly check gas and water taps and light switches after turning them off.

I need things to be arranged in a particular order.

I feel that there are good and bad numbers.

I wash my hands more often and longer than necessary.

I frequently get nasty thoughts and have difficulty in getting rid of them.

**Q28 Tell us how often you have felt each of the following sentiments since the delivery of your baby?**

Not at all Once or twice Sometimes Often, but for >1month Often, for >1 month

Did you have bad dreams of giving birth or of your baby’s hospital stay?

Did you have upsetting memories of giving birth or of your baby’s hospital stay?

Did you have any sudden feelings as though your baby’s birth was happening again?

Did you try to avoid thinking about childbirth or your baby’s hospital stay?

Did you avoid doing things that might bring up feelings you had about childbirth or your baby’s hospital stay (e.g., not watching a TV show about babies)?

Were you unable to remember parts of your baby’s hospital stay?

Did you lose interest in doing things you usually do (e.g., did you lose interest in your work or family)?

Did you feel alone and removed from other people (e.g., did you feel like no one understood you)?

Did it become more difficult for you to feel tenderness or love with others?

Did you have unusual difficulty falling asleep or staying asleep?

Were you more irritable or angry with others than usual?

Did you have greater difficulties concentrating than before you gave birth?

Did you feel more jumpy (e.g., did you feel more sensitive to noise, or more easily startled)?

Did you feel more guilt about the childbirth than you felt you should have felt?

**Q29 Thank you for your responses thus far, we have just a few more questions about your birth experience before you leave us.**

**Q30 Did you experience any of these with the birth of your child? Select all that apply.**

Assisted delivery (vacuum or forceps)

Breech baby

Emergency or unexpected admittance to hospital

Episiotomy

Hemorrhaging

High blood pressure

Long labor

Needed a blood transfusion

Needed to be induced

Unplanned or emergency cesarean section (C-section)

Unplanned epidural / medication

Uterine rupture

Other, please specify: ____________________

None of these

**Q31 Were any of the following used during delivery for pain management? Select all that apply.**

Bathing / soaking in a tub

Breathing exercises

Epidural block

Massage

Music

Narcotics

Spinal block

Tranquilizers

Visualization techniques

Other, please specify: ____________________

None of these

**Q32 Did your baby have any serious health issues at birth?**

No, I had a healthy baby

Yes, but did not require admittance to NICU

Yes, and did require admittance to NICU

*If Yes, and did require admittance to NICU*

**Q33 How long was your baby in the NICU?**

Less than 1 day

1-2 days

3-4 days

5-7 days

8-14 days

2-4 weeks

1 month or longer

**Q34 Did you experience any health issues after delivery?**

Excessive bleeding

Excessive pain

Fever

Hemorrhoids

Incontinence

Infection

Mastitis or breast issues

Postpartum depression

Postpartum preeclampsia

Prolapse (bladder, rectum, uterine)

Other, please specify: ____________________

None of these

**Q35 Describe any experience with breastfeeding and your new baby:**

I currently breastfeed my baby

I have breastfed, but no longer do

I tried unsuccessfully to breastfeed

I have not breastfed my baby

**Q36 How would you describe your weight gain during your recent pregnancy?**

I gained less than the recommended amount

I gained the recommended amount

I gained more than the recommended amount

I am not sure

**Q37 Approximately how much weight would you say you gained during your recent pregnancy?**

None

1-14 pounds

15-24 pounds

25-35 pounds

36-50 pounds

More than 50 pounds

Prefer not to say

**Q38 Finally today, tell us, since giving birth, how often do you do each of the following?**

Every day A few times a week Once a week Less often Never

Drink alcohol

Practice meditation or breathing

Smoke cigarettes

Exercise

Smoke marijuana

Eat healthy

Write in a journal

Use illegal drugs

Practice yoga

Read a book

Participate in an online discussion group

Meet with friends

Eat junk food

Go for walks

*If Cohort 1*

**Q39 Again we thank you wholeheartedly for your voluntary participation in this important research study. Your time and honesty are greatly valued, and we wish you the best on your journey of motherhood. You have earned $50 in Amazon gift cards to be awarded to you immediately in two $25 gift codes. Additionally, you have earned a final entry into the random drawing for a $1000 cash prize. The drawing will take place on or about October 15, 2017 so be on the lookout in your email for notification if you’ve won. Thank you!**

*If Cohort 2*

**Q40 Again we thank you wholeheartedly for your voluntary participation in this important research study. Your time and honesty are greatly valued, and we wish you the best on your journey of motherhood. You have earned $50 in Amazon gift cards to be awarded to you immediately in two $25 gift codes. Additionally, you have earned a final entry into the random drawing for a $1000 cash prize. The drawing will take place on or about April 15, 2017 so be on the lookout in your email for notification if you’ve won. Thank you!**
