# Supplementary material for: Leveraging Digital Technology in Conducting Longitudinal Research on Mental Health in Pregnancy: Longitudinal Panel Survey Study
Source: JMIR Pediatr Parent. 2021 Apr 27;4(2):e16280. doi: 10.2196/16280 (PMC8114159; doi:10.2196/16280)
Supplement: Multimedia Appendix 2 [file pediatrics_v4i2e16280_app2.docx]

**Lessons Learned: Leveraging Digital Technology in Conducting Longitudinal Research on Mental Health in Pregnancy: Longitudinal, Panel Survey Study**

**Multimedia Appendix 2**

**General Interest and Health Questions**

**T2**

**During the past two weeks, how healthy have you been eating? Choose one.**

Extremely healthy

Very healthy

Pretty healthy

Not very healthy

Not at all healthy

**What foods, if any, are you craving? Choose all that apply.**

Sweet

Salty

Healthy

Unhealthy

I don’t crave foods

**Overall, how has your sleep quality been over the past 7 days? Choose one.**

Very good

Good

Fair

Poor

Very poor

**Over the course of the past two weeks, how often do you find yourself taking naps? Choose one.**

A few times a day

Once a day

A few times a week

Once a week

Less than once a week

I’d like to nap, but don’t have the time to do it

**Select the activities in which you have participated in the past 7 days: Choose all that apply.**

Practiced meditation or breathing

Exercised

Ate healthy

Wrote in a journal or blog

Practiced yoga

Read a book

Participated in an online discussion group

Met with friends

Went for a walk

None of these

**T3**

**How much do you enjoy being pregnant so far? Choose one.**

Neither like it nor dislike it

Dislike it most of the time

Hate it and can’t wait for baby to be here!

**Have you been feeling at all anxious about the baby’s health? Choose one.**

Not at all

Not very

Somewhat

Very

**T4**

**To begin today’s short, ten-minute survey, please tell us: was this pregnancy planned? Choose one.**

Yes

No

I don’t feel like sharing

**When did you tell your spouse/partner about your pregnancy? Choose one.**

As soon as I found out

A few hours later

A day later

A few days later

A week or so later

Longer than a week

Other / Not applicable

**When did you share your pregnancy news with everyone (other than your spouse/partner or close family members)? Choose one.**

As soon as I found out

Shortly after I found out

After the first trimester was over

During the second trimester

Later

Haven't yet shared the news

*Answer If To begin today’s short, ten-minute survey, please tell us: was this pregnancy planned? Yes Is Selected*

**How long did it take you to get pregnant? Choose one.**

Less than 3 months

3 to 6 months

6 months to a year

More than a year

**When you need information, guidance, and/or support related to your pregnancy, which of the following do you rely on? Please select all that apply.**

Spouse/partner

Your mother

Other family members

Friends you talk to in person (or via phone/text/IM/email)

Friends you connect with on social media (Facebook, Twitter)

Other women pregnant or moms online (BabyCenter Community, What To Expect Community)

Other women pregnant or moms you meet in person/ Local support groups

Medical professionals (doctors, nurses)

Midwife/doula

Religious groups

Email newsletters

Internet/websites

Mobile apps

Books

Other (please specify) ____________________

None of these

**T5**

**How do you feel about your pregnant body? Choose one.**

Love it

Like it

Neither like nor dislike it

Dislike it

Hate it

**Let’s talk about something different: maternity clothing. How do you feel about maternity clothing? Choose one.**

I love it, it’s comfy and cute

I hate it and look forward to wearing regular clothes again

Neutral, it’s just something that needed during pregnancy

I don’t wear or don’t plan to wear maternity clothing

**Now we would like to learn about your recent sleep habits, as some women experience changes in their sleep habits during pregnancy. Overall, how has your sleep quality been over the past 7 days? Choose one.**

Very good

Good

Fair

Poor

Very poor

**T6**

**We’d like to learn how you feel about some of the things moms-to-be might do for themselves before the baby arrives. How do you feel about babymoons? Choose one.**

They are a fantastic idea!

They are OK for others, but not my cup of tea

They are a total waste of time and money

Not familiar with babymoons

**Would you ever get a tattoo to commemorate your baby? Choose one.**

Yes

No

I already have

**T7**

**We’d like to learn more about any thoughts you have on sleeping arrangements once your baby arrives. Will your baby have its own nursery? Choose one.**

Yes, and it’s finished!

Yes, but it’s a work in progress

Yes, but it hasn’t been started

No, I have other arrangements

**Do you plan to have your baby sleep in your bedroom at home at all? Choose one.**

Yes, that will be the primary place the baby sleeps

Yes, but only for a period of time

No, my baby will always sleep in his/her own space

Not certain, will decide once the baby is here

**Do you feel at all anxious about breastfeeding your baby? Choose one.**

Not at all

Not very

Somewhat

Very

Not able to breastfeed for health reasons

Not planning to breastfeed

**How would you describe the prenatal care you have received from you obstetrician or other health care provider? Choose one.**

Excellent

Good

Fair

Poor

Have not received any prenatal care

**T8**

**Do you know the gender of your baby? Choose one.**

No, it’s too soon to tell

Yes, it’s a girl!

Yes, it’s a boy!

Yes, there are twins (or multiples)!

No, I want it to be a surprise!

**Do you believe any wives’ tales that try to predict a baby’s gender? Choose one.**

Yes, they must be rooted in some fact

No, it’s all nonsense

I’ve never heard any wives’ tales about predicting gender

**We would like to learn about your sleep habits, as some women experience changes in their sleep habits during pregnancy. Overall, how has your sleep quality been over the past 7 days? Choose one.**

Very good

Good

Fair

Poor

Very poor

**T9**

**We’d like to know more about how you feel about baby names: Do you already have a name chosen for your baby? Choose one.**

Yes, the name is decided!

Yes, there are a few names that are front-runners

No, haven’t made any decisions

No, haven’t thought about it at all

**When do you think it’s appropriate to share a baby name with family and friends? Choose one.**

As soon as the name is chosen

As the baby’s due date nears

After the baby is born

**T10**

**We’d like to learn about your thoughts on baby gifts and baby showers. Have you had or do you plan to have a baby shower?**

Yes, I’ve already had a shower

Yes, planning on having a shower

I had a shower for a previous baby/pregnancy, but not planning one for this pregnancy

No, no plans for this

**Should dads and other male friends and family be invited to baby showers?**

Yes, definitely!

Maybe, depending on the circumstances

No

**Have you set up a baby registry or do you plan to do so?**

Yes, I want to make sure the gifts I receive are what I want

Yes, it’s just easier for everyone if there is a list to pick from

No, it’s tacky to ask people to purchase specific gifts

No, I wouldn’t expect anyone to buy gifts

*Answer If Cohort Is Equal to 1*

**Let us know if you have you experienced any of the following during this pregnancy? Please select all that apply.**

Back pain

Depression

Difficulty eating (e.g., motivation to eat healthy, appetite)

Difficulty managing your weight

Difficulty sleeping

Fatigue or lack of energy

Gestational diabetes

Headaches or migraines

Insomnia or trouble sleeping

Mood swings

Morning sickness

Nausea

Preeclampsia (high blood pressure)

Other (please specify) ____________________

None of these

**When in your pregnancy did you have your first visit with an obstetrician or other health care provider? Choose one.**

Weeks 4…

Weeks 35+

Have not had an appointment

**How would you describe the prenatal care you have received from you obstetrician or other health care provider? Choose one.**

Excellent

Good

Fair

Poor

Have not received any prenatal care

*Answer If Cohort Is Equal to 1*

**How would you describe your weight gain during this pregnancy?**

I am gaining less than the recommended amount

I am gaining the recommended amount

I am gaining more than the recommended amount

I am not sure

**T12**

**To begin, we’d like to know a little about what the birth experience was like for you: Did you deliver vaginally or have a C-section? Choose one.**

Vaginally

C-section

**Did any of the following occur for you or your baby during labor or delivery? Please select all that apply.**

Baby was induced

Labor was very long

Baby was born early (before 37 weeks)

Baby had to be delivered via emergency C-section

Baby was delivered using forceps or suction

Baby went to NICU following delivery

Other (please specify) ____________________

None of these

Prefer not to answer

**T13**

**Finish this statement: Life with my new baby is:**

Easier than I expected

About what I expected

Harder than I expected

An adventure! I didn’t know what to expect.

**Does your baby sleep in your bedroom at home? Choose one.**

Yes, and will so for the foreseeable future

Yes, but only for a period of time

No, the baby will always sleep in his/her own space

**How well does your baby sleep overall? Choose one.**

Very well

As well as expected for a newborn

Not at all well

**T14**

**We’d like to learn a little about how your baby has been feeding. How would you describe your experience with baby’s feeding?**

Excellent – baby feeds very well

Good – there have been a few challenges

Poor – it’s been a struggle

**Have you experienced any of the following issues with your baby? Please select all that apply.**

Colic

Gastroesophageal reflux

Unable to breastfeed baby for health reasons

Baby refusing to breastfeed

Baby allergic to formula

Baby refusing to take the bottle

Concerns about milk supply

Baby not latching properly

Baby wanting to breastfeed all the time

Other (please specify) ____________________

None

**Have you had a postpartum check-up with your doctor or obstetrician? Choose one.**

Yes, I’ve been to my appointment

No, but I have one scheduled

No, I don’t plan to

**How would you describe the postnatal care you have received from you obstetrician or other health care provider after the birth of your baby? Choose one.**

Excellent

Good

Fair

Poor

Have not received any prenatal care

**T15**

**We’d like you to think back to your pregnancy for a moment: At what point in your pregnancy do you feel like you “started to show”? Choose one.**

Month - 1

Month - 2

Month - 3

Month - 4

Month - 5

Month - 6

Month - 7

Month - 8

Never

**What do you miss most about being pregnant? Choose one.**

The closeness with my baby

The anticipation and preparations

The special attention paid to me

The comfy maternity clothing

Nothing! I don’t miss being pregnant

**Did you ever personally contact your health insurance provider when you were pregnant for any of the following reasons? Please select all that apply.**

To notify them of my pregnancy

To inquire about pregnancy benefits

To check on the status of a claim

To look into costs/reimbursements for medical services

To look into costs/reimbursements for birth/delivery

To inquire about a free/discounted breast pump

To inquire about free/discounted childbirth classes

Other (please specify) ____________________

No, I never contacted them
