# Supplementary material for: Leveraging Digital Technology in Conducting Longitudinal Research on Mental Health in Pregnancy: Longitudinal Panel Survey Study
Source: JMIR Pediatr Parent. 2021 Apr 27;4(2):e16280. doi: 10.2196/16280 (PMC8114159; doi:10.2196/16280)

**Lessons Learned: Leveraging Digital Technology in Conducting Longitudinal Research on Mental Health in Pregnancy: Longitudinal, Panel Survey Study**

**Multimedia Appendix 3**

**Janssen/BabyCenter Study Obsessive Compulsive Inventory Factor Structure: Cohort 1 (n=475)**

| **Promax Rotated Factor Pattern (Standardized Regression Coefficients)** | | Ordering | Obsessing | Hoarding | Checking | Washing | Neutralizing |
| --- | --- | --- | --- | --- | --- | --- | --- |
| OCR_3 | I get upset if objects are not arranged properly. | **76*** | -2 | -5 | 15 | -3 | 12 |
| OCR_9 | I get upset if others change the way I have arranged things. | **73*** | 10 | 9 | 3 | 8 | -9 |
| OCR_15 | I need things to be arranged in a particular order. | **63*** | -6 | 4 | 5 | 23 | 10 |
| OCR_12 | I am upset by unpleasant thoughts that come into my mind against my will. | 0 | **78*** | 7 | 9 | 9 | -10 |
| OCR_18 | I frequently get nasty thoughts and have difficulty in getting rid of them. | -7 | **71*** | -2 | 9 | 11 | 13 |
| OCR_6 | I find it difficult to control my own thoughts. | 11 | **69*** | 2 | -7 | 3 | 7 |
| OCR_7 | I collect things I don't need. | 1 | 1 | **86*** | 4 | -8 | 1 |
| OCR_13 | I avoid throwing things away because I am afraid I might need them later. | 4 | -1 | **78*** | -7 | 20 | -5 |
| OCR_1 | I have saved up so many things that they get in the way. | 3 | 11 | **40*** | 22 | -26 | 18 |
| OCR_2 | I check things more often than necessary. | 15 | 10 | -1 | **70*** | -9 | 4 |
| OCR_8 | I repeatedly check doors, windows, drawers, etc. | 10 | -1 | 5 | **66*** | 30 | -14 |
| OCR_14 | I repeatedly check gas and water taps and light switches after turning them off. | -15 | -4 | 22 | **41*** | 29 | 29 |
| OCR_5 | I find it difficult to touch an object when I know it has been touched by strangers or certain people. | 23 | 12 | -4 | -17 | **65*** | 13 |
| OCR_11 | I sometimes have to wash or clean myself simply because I feel contaminated. | 10 | 24 | -1 | 15 | **61*** | -8 |
| OCR_17 | I wash my hands more often and longer than necessary. | -4 | -3 | -8 | 38 | **61*** | 15 |
| OCR_16 | I feel that there are good and bad numbers. | 4 | 5 | 7 | -14 | 17 | **74*** |
| OCR_4 | I feel compelled to count while I am doing things. | 11 | 0 | -5 | 21 | -1 | **64*** |
| OCR_10 | I feel I have to repeat certain numbers. | 9 | 19 | 21 | 21 | 15 | **34*** |

**The Obsessive-Compulsive Inventory: development and validation of a short version. Psychol Assess. 2002 Dec;14(4):485-96. PMID: 12501574**

**Foa EB, Huppert JD, Leiberg S, Langner R, Kichic R, Hajcak G, Salkovskis PM**


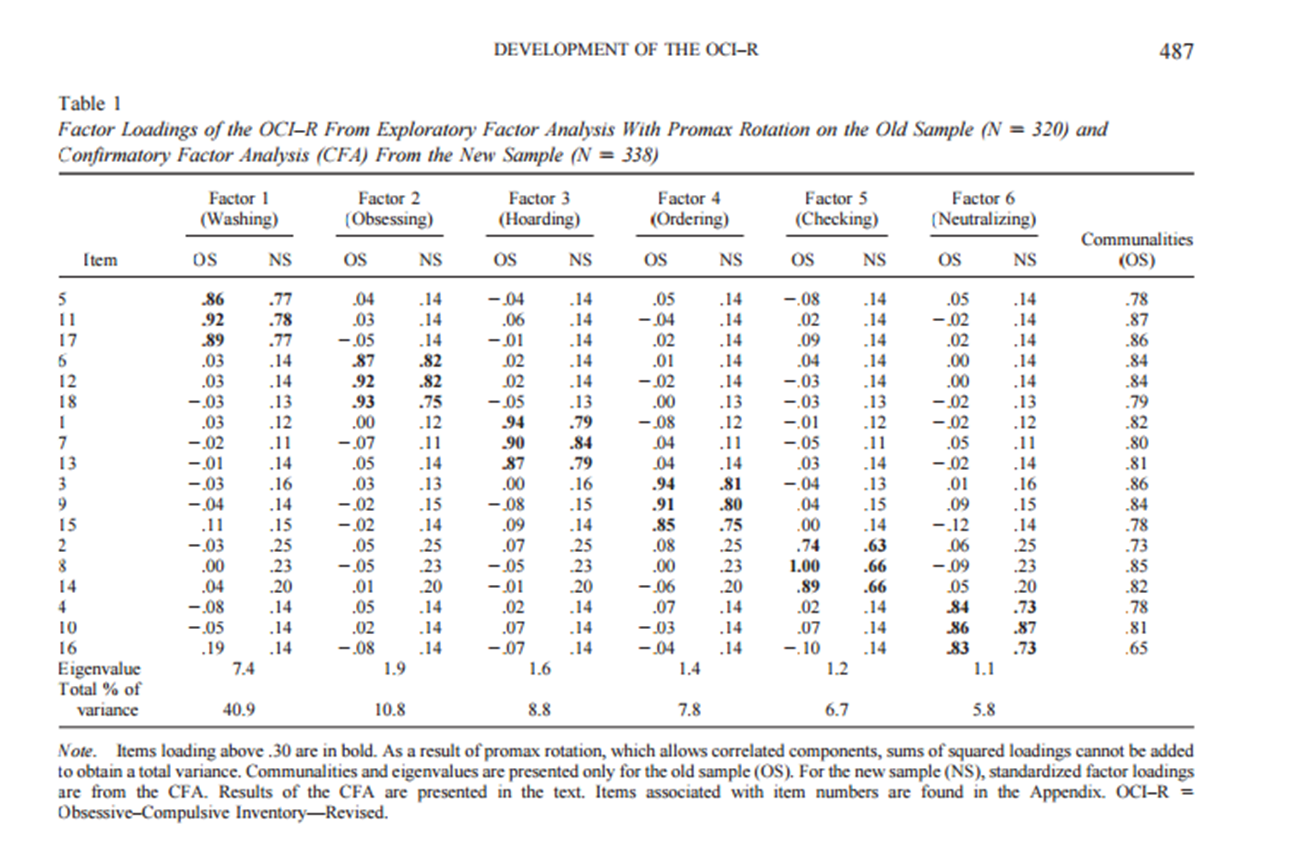

Supplement: Multimedia Appendix 3 [file pediatrics_v4i2e16280_app3.docx]
